# Predictive Modeling of Preoperative Sleep Disorder Risk in Older Adults by Using Data From Wearable Monitoring Devices: Prospective Cohort Study

**DOI:** 10.2196/79008

**Published:** 2026-02-11

**Authors:** Jingjing Li, Binxu Yang, Puzhong Gao, Dan Feng, Xinxin Shao, Xusihong Cai, Shuwen Huang, Yu Huang, Qingde Wa, Jing Zhou

**Affiliations:** 1 The Second Affiliated Hospital of ZunYi Medical University Zunyi, Guizhou China; 2 School of Nursing Zunyi Medical University Zunyi, Guizhou China

**Keywords:** preoperative sleep disorder, older adults, smart wearable devices, risk prediction model, logistic regression

## Abstract

**Background:**

Sleep disorders are common among older adults undergoing surgery and contribute significantly to postoperative complications, delayed recovery, and higher health care costs. The combined effects of age-related physiological changes and surgical stress further disrupt sleep in this vulnerable group. However, current tools for predicting surgical risk rarely account for the specific physiological, clinical, and psychological factors that affect older patients. While wearable devices are used to monitor sleep, most prediction models focus on general sleep quality in nonsurgical populations, leaving a gap in forecasting preoperative sleep disorders in older surgical candidates. Therefore, we developed and validated a tailored risk prediction model that integrates objective sleep data from wearable devices with comprehensive clinical and psychosocial evaluations for older adults preparing for surgery.

**Objective:**

We aimed to develop and validate a risk prediction model for preoperative sleep disorders in older adult surgical patients by using data from smart wearable devices and clinical assessments, thereby facilitating early identification of the influencing factors and providing a scientific basis for personalized care planning.

**Methods:**

We conducted a prospective study at the Second Affiliated Hospital of Zunyi Medical University. A cohort of 242 older surgical patients was monitored using smart rings on the night before surgery. We simultaneously collected data on sociodemographic factors, cognition, and psychological status. As per preoperative sleep assessments, patients were classified into sleep disorder and non–sleep disorder groups. Independent predictors of sleep disorders were identified using univariable and multivariable logistic regression. These predictors were used to build a risk prediction model, which was internally validated with 1000 bootstrap samples. The model’s performance was evaluated by its ability to discriminate between groups (using receiver operating characteristic curves), its calibration, and its clinical usefulness (via decision curve analysis).

**Results:**

Multifactorial logistic regression analysis showed that Hospital Anxiety and Depression Scale score (odds ratio [OR] 3.21, 95% CI 1.54-6.69; *P*=.002), number of awakenings (OR 3.33, 95% CI 1.82-6.12; *P*<.001), duration of rapid eye movement sleep (OR 0.96, 95% CI 0.93-0.99; *P*=.04), and duration of light sleep (OR 0.98, 95% CI 0.96-0.99; *P*=.01) were independent risk factors for preoperative sleep disturbances in older adults (*P*<.05). The receiver operating characteristic curve showed an area under the curve of 0.92, and the calibration curve indicated good model calibration. Decision curve analysis showed that the model improved the maximum net benefit across risk thresholds ranging from 0.2 to 0.8, indicating high clinical utility.

**Conclusions:**

The risk prediction model developed using smart ring–derived data effectively identifies older adult surgical patients at elevated risk of preoperative sleep disturbances, thereby facilitating timely and individualized interventions. This advancement provides a robust scientific foundation for delivering personalized perioperative care, with the potential to improve postoperative outcomes and alleviate the health care burden in this vulnerable population.

## Introduction

### Background

Sleep disorders, characterized by disruptions in sleep duration, sleep quality, and circadian rhythm [1], are frequently accompanied by anxiety, depression, and cognitive impairment, collectively exerting detrimental effects on patients’ perceptions and behaviors [2]. With advancing age, older adults typically exhibit characteristic alterations in sleep architecture, including prolonged sleep latency, reduced total sleep time, and increased sleep fragmentation [3]; perioperative factors may further exacerbate these disturbances [4]. Intraoperative administration of anesthetics and analgesics can disrupt neurotransmitter homeostasis—particularly involving dopamine, norepinephrine, and γ-aminobutyric acid (GABA)—in older adults, resulting in heightened sensitivity to external stimuli and increased nocturnal arousals [5-7].

With the global population aging at an unprecedented rate, the demand for geriatric surgical procedures continues to grow annually. According to the World Health Organization, individuals older than 65 years now account for more than 40% of all surgical interventions worldwide [8]. This demographic is typically marked by physiological aging, multisystem functional deterioration, and a substantial burden of comorbidities, which collectively lead to increased perioperative vulnerability and a postoperative complication rate 2 to 3 times higher than that observed in younger cohorts [9]. Notably, sleep disturbances constitute a critical factor influencing postoperative recovery in older adults [2]. The reported incidence of perioperative sleep disturbances in older adults ranges from 15% to 84% [10,11]. Approximately 23% of the patients experience postoperative sleep disorders that may persist for up to 4 days, and by postoperative day 15, approximately 25% continue to exhibit pronounced symptoms of sleep deprivation. A subset of these patients still requires pharmacological intervention to maintain sleep continuity [12].

Perioperative sleep disturbances in older adults not only elevate the risk of postoperative delirium [13] but are also linked to a spectrum of adverse outcomes, including an increased risk of stroke, compromised immune function, elevated obesity risk, and higher morbidity and mortality rates [14,15]. Specific disturbances such as insomnia and circadian rhythm disruption during the perioperative period can result in daytime fatigue and a heightened risk of falls in older adults, thereby delaying wound healing, prolonging hospitalization, and contributing to increased health care expenditures and elevated mortality risk [13,16,17]. One survey indicated that, over a 6-month period, health care costs were approximately US $1100 higher for older adults who developed sleep disorders than for those without such conditions [17]. The economic burden of sleep disorders is substantial, amounting to 0.7% of Australia’s gross domestic product, while nonfinancial costs account for an additional 3.2% of the national disease burden [18]. The presence of perioperative sleep disorders in older adults not only impairs sleep quality, physical health, and postoperative recovery but also exerts a profound impact on long-term quality of life [19]. Therefore, early identification and timely management of sleep disturbances in older adult surgical patients are crucial for improving clinical outcomes and alleviating the broader societal health care burden.

With the rapid advancement of information technology, smart wearable devices—such as smart bracelets and smartwatches—have demonstrated significant potential in monitoring individual sleep patterns. Evidence suggests that smart wearable devices yield measurements comparable to polysomnography in terms of total sleep time, nocturnal awakenings, and sleep efficiency [20]. Furthermore, sleep metrics obtained from smart rings exhibit good concordance with assessments derived from the Pittsburgh Sleep Quality Index (PSQI) [21]. Smart rings detect micromovements via a triaxial accelerometer and demonstrate high sensitivity and specificity in tracking parameters such as sleep onset latency, wake time, total sleep duration, rapid eye movement (REM) sleep, and the number of nocturnal awakenings [22].

Risk prediction models are designed to estimate the likelihood of future clinical events, such as the onset of a specific disease in a given patient population [23]. The identification and management of sleep disturbances in surgical settings can enhance perioperative care, reduce postoperative complications and associated health care expenditures, and improve patient throughput efficiency [24]. Although several risk prediction models for sleep disorders in older adults have been proposed [24,25], their predictive performance remains inconsistent, and none has been specifically designed for older adults undergoing surgery. Moreover, although recent studies have begun to use smart wearable devices for sleep monitoring—for example, machine learning–based approaches that forecast sleep efficiency hours before bedtime [26]—these efforts have largely focused on general sleep quality metrics in nonsurgical populations. Building on this work, this study extends the application of wearable technology by developing a targeted risk prediction model for preoperative sleep disorders in older adults undergoing surgery. By integrating high-resolution, objective sleep architecture data captured by a smart ring with key clinical and psychological assessments, the model addresses the distinct physiological and psychological vulnerabilities of this patient population. This approach represents a substantial advance beyond simple sleep efficiency forecasting, enabling proactive identification of clinically meaningful sleep disorders in the high-risk perioperative setting.

### Objective

This study aimed to construct a predictive nomogram by integrating sleep-related data collected via smart wearable devices from older adult surgical patients to estimate the risk of postoperative sleep disturbances and support the early identification and management of high-risk individuals.

## Methods

This study was designed and executed in accordance with the Transparent Reporting of a Multivariable Prediction Model for Individual Prognosis or Diagnosis (TRIPOD) guidelines [27,28].

### Study Design and Participants

This prospective cohort study enrolled older adults who underwent elective surgical procedures between August 1, 2023, and January 30, 2024, at the Second Affiliated Hospital of Zunyi Medical University, China. Inclusion criteria were as follows: (1) aged between 60 and 80 years, (2) scheduled for elective surgery, (3) had an American Society of Anesthesiologists physical status of I to III, (4) fully conscious and capable of effective communication, and (5) had no documented history of sleep disorders. Exclusion criteria were as follows: (1) presence of central nervous system diseases or psychiatric disorders, (2) a history of sedative or hypnotic medication use, (3) undergoing daytime surgery or neurosurgical procedures, (4) New York Heart Association class IV heart failure, and (5) planned postoperative admission to the intensive care unit.

### Data Collection

Patient clinical data were extracted from electronic medical records and included variables such as age, sex, diagnosis, BMI, and surgical specialty department. Additionally, sleep-related parameters were acquired via wearable monitoring devices, encompassing sleep onset time, wake-up time, total sleep time, durations of deep and light sleep, REM sleep duration, and the number of awakenings. Furthermore, several validated instruments were administered, including the PSQI, Hospital Anxiety and Depression Scale (HADS), Mini-Mental State Examination (MMSE), and Numerical Rating Scale (NRS) for pain assessment.

Biochemical markers were also obtained, including white blood cell count; neutrophil count; red blood cell count; hemoglobin; hematocrit; platelet count; prothrombin time; activated partial thromboplastin time; fibrinogen, serum potassium, sodium, and calcium levels; alanine aminotransferase; aspartate aminotransferase; prealbumin; blood urea nitrogen; creatinine; blood glucose; total protein; cholinesterase; high-sensitivity C-reactive protein; and creatine kinase and its isoenzymes.

**Figure 1:**
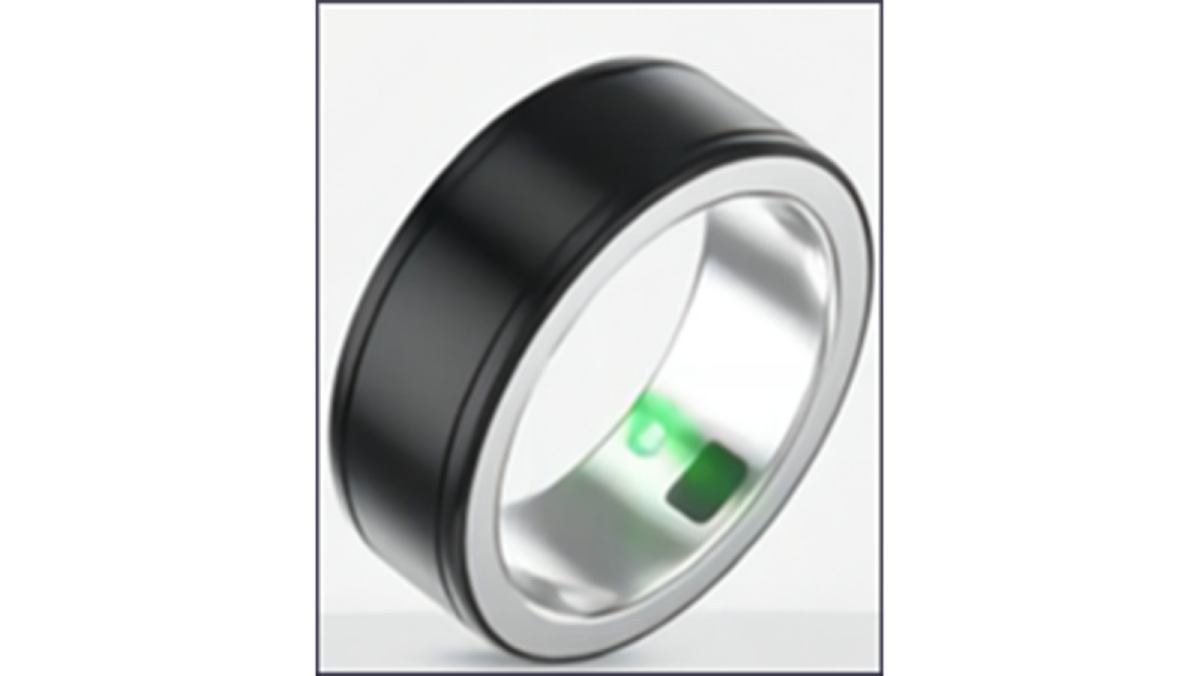
SRing2 smart ring.

In this study, the PSQI was used to evaluate the sleep quality of older adults on the night preceding surgery. Concurrently, a smart ring (Figure 1; SRing2; Shenzhen Century Modern Technology Development Co, Ltd) was used to monitor sleep parameters in real time, including sleep onset time, wake-up time, total sleep duration, REM sleep duration, durations of light and deep sleep, and the number of awakenings. The implementation procedure was as follows: the smart ring was uniformly fitted on each patient by the researcher before 10:00 PM on the night before surgery and removed upon waking up the following morning. Thus, this smart ring–based monitoring technology provided more objective and comprehensive sleep data.

### Sleep Quality Grouping

On the basis of the PSQI scores and sleep data collected via the smart ring, in conjunction with established diagnostic criteria for sleep disorders, 242 older adults were classified into 2 groups: the sleep disorder group, defined by PSQI scores of 7 points or more [29] and total sleep duration of less than 6.5 hours [30], and the non–sleep disorder group, characterized by PSQI scores of 7 points or less and total sleep duration of 6.5 hours or more.

### Ethical Considerations

This study was approved by the ethics committee of the Second Affiliated Hospital of Zunyi Medical University (KYLL-2023-012). Written informed consent was obtained from all participants before enrollment. Participants were informed of their right to withdraw from the study at any time without penalty. All personal data collected from the devices were deidentified before analysis. The research data are encrypted and stored on secure servers, accessible only to members of the research team. No financial compensation was provided to the participants.

### Statistical Analysis

All statistical analyses were conducted using R (version 4.4.1; R Foundation for Statistical Computing). Normally distributed continuous variables were summarized as means (SDs) and compared between groups using Student *t* test (2-tailed). Nonnormally distributed variables were presented as medians (IQRs) and compared using the Mann-Whitney U test. Categorical variables were expressed as frequencies and percentages and compared using the chi-square test or Fisher exact test, as appropriate.

Candidate predictors included sociodemographic characteristics (age, sex, and BMI), comorbidities, surgical specialty, biochemical markers, psychometric scale scores (PSQI, HADS, MMSE, and NRS), and sleep parameters derived from the smart ring (including total sleep time, REM sleep duration, light sleep duration, and the number of awakenings). Variable selection was conducted using the least absolute shrinkage and selection operator regression with 10-fold cross validation. Variables with nonzero coefficients in the least absolute shrinkage and selection operator model, together with variables with *P*<.10 in univariable logistic regression or judged to be clinically relevant, were entered into a multivariable logistic regression model. The final model was constructed using backward stepwise selection based on the Akaike information criterion, identifying the HADS score, number of awakenings, REM sleep duration, and light sleep duration as independent predictors. These predictors were incorporated into the nomogram.

Model performance was evaluated using receiver operating characteristic (ROC) curves, calibration plots, and decision curve analysis (DCA). Internal validation was conducted using 1000 bootstrap resamples to estimate optimism-corrected performance metrics (eg, area under the curve [AUC]) and assess the stability of the variable selection process. A 2-sided *P* value of <0.05 was considered statistically significant.

### Model Evaluation

The predictive performance of the model was evaluated using ROC curve analysis, with the AUC representing the model’s discriminatory ability [31]. DCA was used to assess the clinical utility and effectiveness of the model. DCA quantifies the net benefit of a predictive model by incorporating threshold probabilities that balance the relative harms of false-positive and false-negative classifications. In recent years, DCA has gained recognition as a superior approach to ROC curve analysis for evaluating the clinical value of predictive models [32]. Therefore, this study used DCA to evaluate the clinical benefit of the model.

## Results

### General Characteristics

A total of 242 older adults were enrolled in this study, comprising 129 (53.3%) female individuals and 113 (46.7%) male individuals. The mean age was 67.45 (SD 6.23) years, and the mean BMI was 23.34 (SD 3.69) kg/m^2^. Regarding ward accommodation, 204 patients (84.3%) were admitted to multioccupancy rooms, while 38 (15.7%) patients stayed in single-occupancy rooms. The average length of hospitalization was 12.52 (SD 5.99) days. Additional details are presented in Table 1.

**Table 1:** Baseline characteristics of older adults (N=242)a.

Variable			Total patients (N=242)		Patients in the non–sleep disorder group (n=202)		Patients in the sleep disorder group (n=40)		Statistics			*P* value	
Age (y), mean (SD)			67.45 (6.23)		67.40 (6.25)		67.70 (6.22)		–0.28^b^			.78	
**S** **ex** **, n (%)**										0.21^c^			.65
	Female	129 (53.3)		109 (54)		20 (50)							
	Male	113 (46.7)		93 (46)		20 (50)							
BMI (kg/m^2^), mean (SD)			23.34 (3.69)		23.31 (3.63)		23.47 (4.06)		–0.24^b^			.81	
**Wardroom type, n (%)**										6.31^d^			.01
	Multioccupancy room	204 (84.3)		165 (81.7)		39 (97.5)							
	Single-occupancy room	38 (15.7)		37 (18.3)		1 (2.5)							
Length of hospital stay (d), mean (SD)			12.52 (5.99)		12.65 (6.16)		11.82 (5.04)		0.80^b^			.43	
**Residence, n (%)**										2.82^c^			.09
	Urban	128 (52.9)		102 (50.5)		26 (65)							
	Rural	114 (47.1)		100 (49.5)		14 (35)							
**Smoking history, n (%)**										2.57^c^			.11
	No	154 (63.6)		133 (65.8)		21 (52.5)							
	Yes	88 (36.4)		69 (34.2)		19 (47.5)							
**Drinking history, n (%)**										1.13^c^			.29
	No	174 (71.9)		148 (73.3)		26 (65)							
	Yes	68 (28.1)		54 (26.7)		14 (35)							
**Diabetes, n (%)**										0.20^c^			.66
	No	216 (89.3)		179 (88.6)		37 (92.5)							
	Yes	26 (10.7)		23 (11.4)		3 (7.5)							
**Hypertension, n (%)**										1.34^c^			.25
	No	169 (69.8)		138 (68.3)		31 (77.5)							
	Yes	73 (30.2)		64 (31.7)		9 (22.5)							

^a^Continuous variables are presented as mean (SD), while categorical variables are expressed as counts and percentages (n, %).

^b^Independent 2-sample *t* test.

^c^Chi-square test.

^d^Fisher exact test.

### Clinical and Sleep-Related Characteristics

Among the 242 older adults enrolled in this study, the mean PSQI score was 7.31 (SD 2.38), the HADS score was 7.21 (SD 1.84), the NRS score for pain was 1.83 (SD 1.07), and the MMSE score was 20.45 (SD 4.65). Sleep parameters included total sleep duration (mean 473.36, SD 146.06 min), REM sleep duration (mean 84.25, SD 39.42 min), deep sleep duration (mean 128.50, SD 59.70 min), light sleep duration (mean 262.57, SD 92.63 min), and the number of awakenings (mean 2.68, SD 1.77; see Multimedia Appendix 1).

### Factors Associated With Sleep Disorders

#### Univariable Analysis

On the basis of the clinical data, older adults’ preoperative sleep status was classified into 2 groups: those with and those without sleep disorders. One-way ANOVA revealed statistically significant differences between the 2 groups in terms of wardroom type, HADS score, NRS score, number of awakenings, total sleep duration, REM sleep duration, deep sleep duration, and light sleep duration (all *P*<.05; Table 2).

**Table 2:** Univariable logistic regression analysis.

Variable			β coefficient (SE)		*z* score		*P* value^a^		Odds ratio (95% CI)
**Wardroom type**									
	Multioccupancy room	2.17 (1.03)		2.11		.04		8.75 (1.16-65.71)	
	Single-occupancy room	—^b^		—		—		1.00 (reference)	
**Psychometric scale scores**									
	Hospital Anxiety and Depression Scale score	1.00 (0.16)		6.35		<.001		2.72 (2.00-3.70)	
	Numerical Rating Scale score	1.22 (0.21)		5.80		<.001		3.38 (2.24-5.11)	
**Sleep parameters**									
	Number of awakenings	0.72 (0.12)		6.01		<.001		2.06 (1.63-2.60)	
	Total sleep duration (min)	–0.02 (0.00)		–6.32		<.001		0.98 (0.97-0.98)	
	Rapid eye movement sleep duration (min)	–0.04 (0.01)		–5.32		<.001		0.96 (0.94-0.97)	
	Deep sleep duration (min)	–0.02 (0.00)		–5.21		<.001		0.98 (0.97-0.99)	
	Light sleep duration (min)	–0.03 (0.00)		–6.10		<.001		0.97 (0.96-0.98)	

^a^*P*<.05 denotes statistical significance.

^b^Not applicable (because this category serves as the reference for comparison).

#### Multivariable Analysis

Multivariable logistic regression was conducted with the presence of sleep disorders as the dependent variable and variables found to be statistically significant in the univariable analysis as independent predictors. The results demonstrated that HADS score (odds ratio [OR] 3.21, 95% CI 1.54-6.69), number of awakenings (OR 3.33, 95% CI 1.82-6.12), duration of REM sleep (OR 0.96, 95% CI 0.93-0.99), and duration of light sleep (OR 0.98, 95% CI 0.96-0.99) were independent predictors of preoperative sleep disorders in older surgical adult patients (all *P*<.05; Table 3).

**Table 3:** Multivariable logistic regression analysis.

Variable	β coefficient (SE)	*z* score	*P* value	Odds ratio (95% CI)
Hospital Anxiety and Depression Scale score	1.17 (0.38)	3.10	.002	3.21 (1.54-6.69)
Number of awakenings	1.20 (0.31)	3.89	<.001	3.33 (1.82-6.12)
Rapid eye movement sleep duration	–0.04 (0.02)	–2.10	.04	0.96 (0.93-0.99)
Light sleep duration	–0.02 (0.01)	–2.52	.01	0.98 (0.96-0.99)

### Development and Validation of the Nomogram-Based Prediction Model

#### Construction of the Nomogram Prediction Model

**Figure 2:**
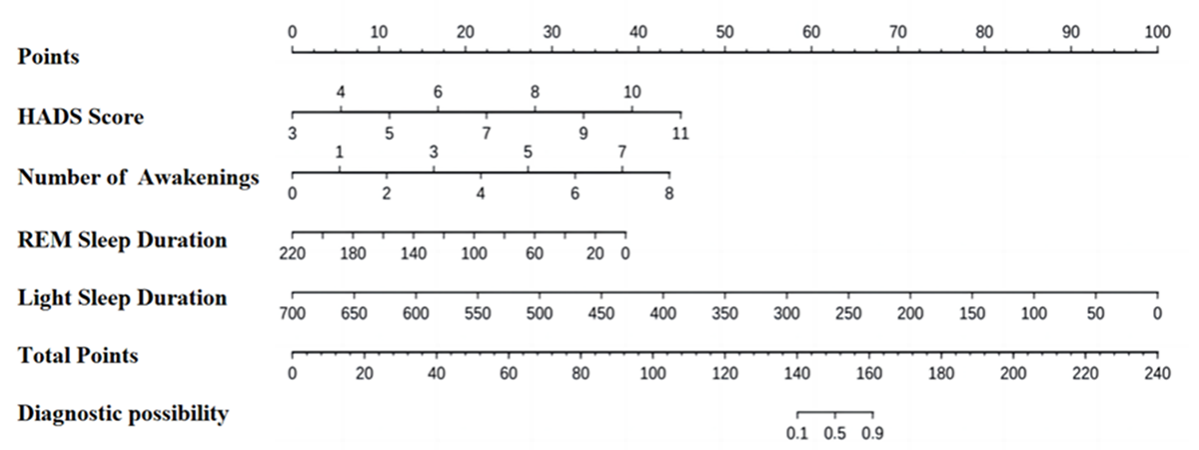
Nomogram for predicting the risk of sleep disorders in older adults undergoing elective surgery. HADS: Hospital Anxiety and Depression Scale; REM: rapid eye movement.

A nomogram was developed based on the results of multivariable logistic regression analysis. As illustrated in Figure 2, each predictor variable is assigned a corresponding point value by projecting vertically onto the scoring axis. The sum of these values yields a total score, which is then mapped onto the total score axis. The final projected value on the risk axis indicates the estimated probability of preoperative sleep disorders in older adult surgical patients.

#### Evaluation of the Predictive Performance of the Nomogram Model

The predictive performance of the nomogram model was evaluated using discrimination, calibration, and DCAs, as mentioned subsequently.

**Figure 3:**
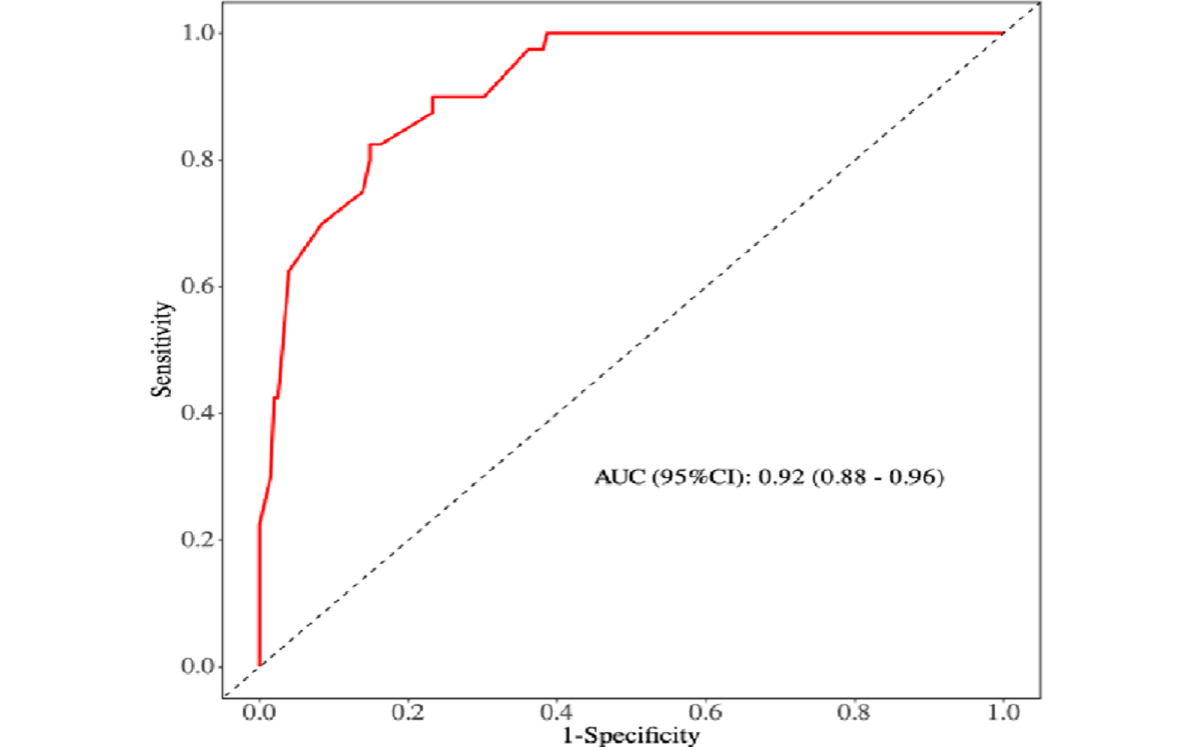
Receiver operating characteristic curves for the prediction model of preoperative sleep disorders in older adults. AUC: area under the curve.

First, internal validation of the nomogram model was conducted using 1000 bootstrap resamples, resulting in an area under the ROC curve of 0.92 (95% CI 0.88-0.96), indicating excellent discriminative ability of the model (Figure 3).

**Figure 4:**
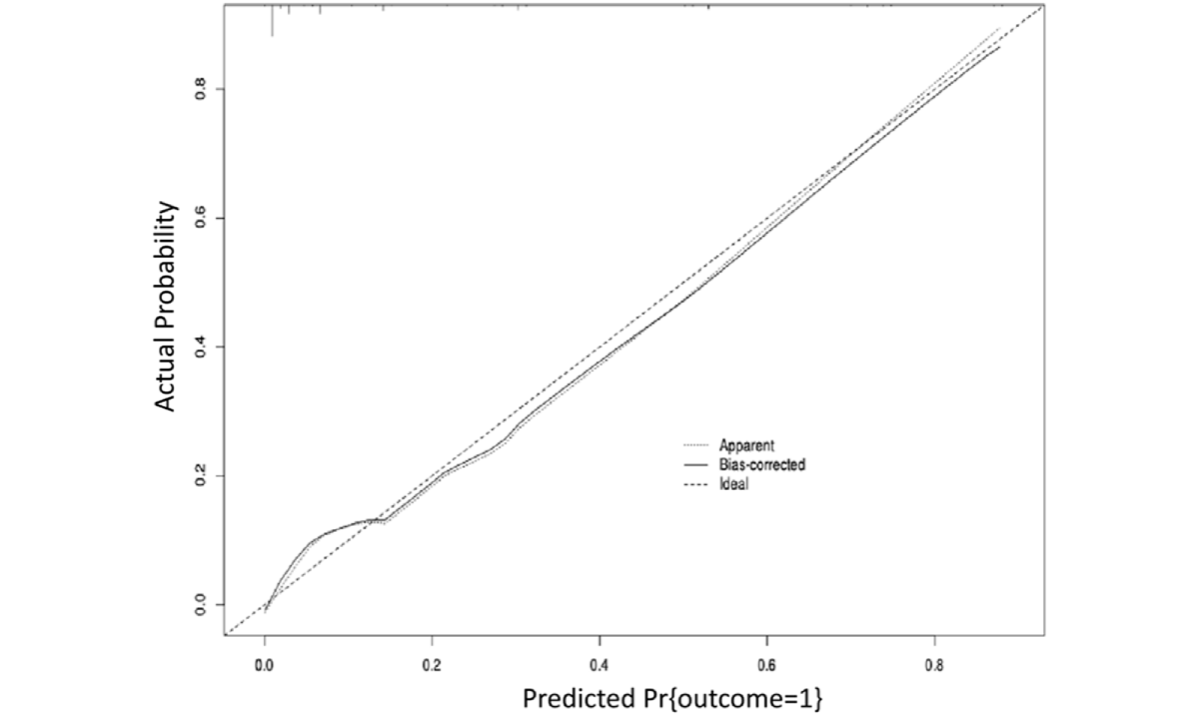
Calibration curve for the risk prediction model of preoperative sleep disorders in older adults.

Second, calibration curves were used to evaluate the agreement between predicted and observed probabilities of sleep disorder onset. The model’s predicted probabilities exhibited minimal deviation from the observed outcomes, indicating good calibration and strong predictive performance (Figure 4).

**Figure 5:**
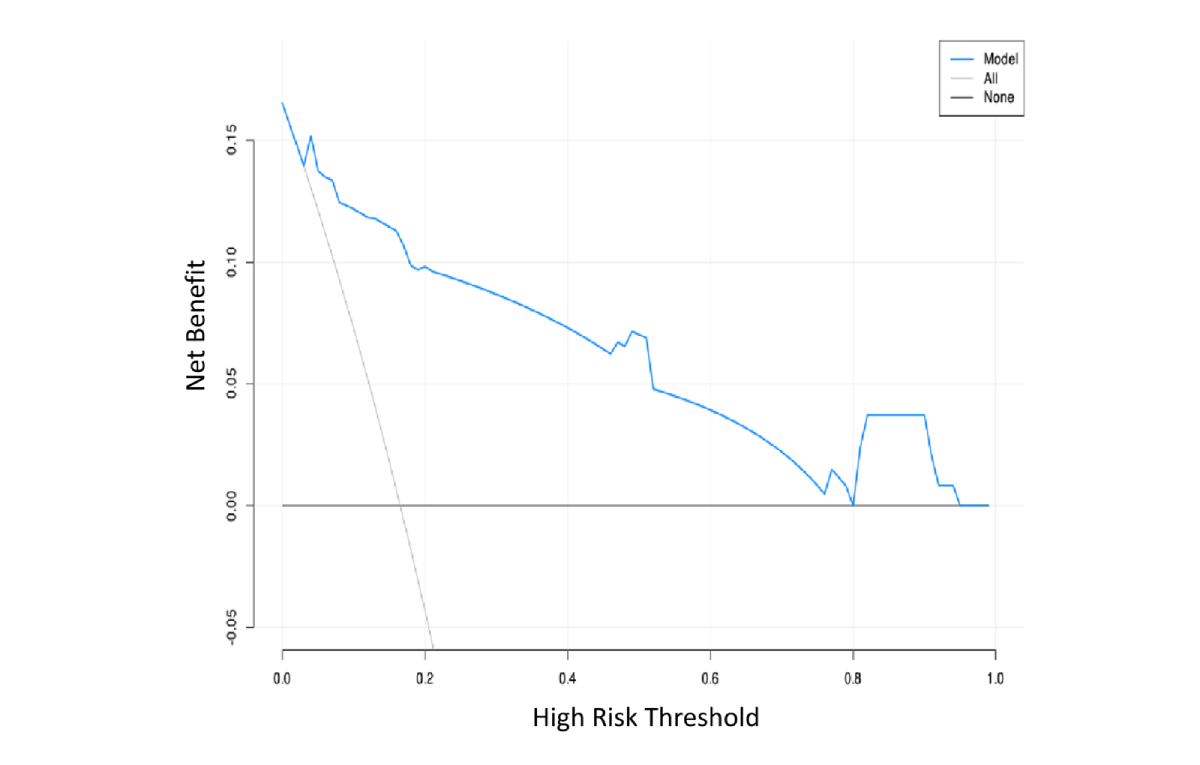
Decision curve analysis of the prediction model for preoperative sleep disorder risk in older adults.

Third, DCA was performed to evaluate the clinical utility of the model by quantifying the net benefit across a range of risk thresholds, in comparison to the default strategies of treating all patients (gray line: “all”) or treating none (black line: “none”). The DCA curve lay above both the “none” and “all” lines across most threshold probabilities, indicating superior predictive performance. The model achieved the greatest net benefit within a threshold probability range of 0.2 to 0.8 (Figure 5).

## Discussion

### Significance of the Prospective Study Design

The prospective design of this study establishes a methodologically robust framework for investigating sleep disorders in the preoperative setting. By systematically assessing key predictors—encompassing psychological state (via HADS scores) and objective sleep architecture parameters (such as the frequency of nocturnal awakenings and the durations of REM and light sleep)—before surgical intervention, this methodology delineates a clear temporal sequence, thereby strengthening causal inference. This design effectively mitigates recall bias and substantiates the role of these factors as antecedent risk determinants rather than mere correlates of postoperative sleep disturbance. Therefore, this design was crucial to accurately quantify the independent contribution of each predictor and reliably construct the subsequent multivariate predictive model.

### Analysis of Factors Affecting Preoperative Sleep Disorders in Older Adults

Anxiety and depression are common in older adult surgical patients, often linked to factors such as previous medical history, changes in the sleep environment, levels of family support, and personal coping styles [33,34]. A strong link exists between these mood states and sleep; 70% to 90% of the patients with anxiety or depression report sleep disturbances [35], a prevalence that is particularly high in older populations [36]. Our results indicate that each 1-unit increase in HADS score was associated with a 3.21-fold higher risk of preoperative sleep disorders in older adults, consistent with previous findings [37,38]. Anxiety and depressive mood have been shown to dysregulate neurotransmitter systems, including serotonin and norepinephrine, in older adults, thereby disrupting sleep-wake regulation and promoting sleep disorders [39]. Critically, anxiety, depression, and poor sleep engage in a bidirectional, vicious cycle, wherein each worsens the others [40]. A state of hyperarousal, driven by dysregulation of neurotransmitters such as GABA and acetylcholine, is a shared pathological feature of both anxiety and sleep disorders [41]. Older adult surgical patients with anxiety and depression exhibit elevated levels of adrenocorticotropic hormone–releasing hormone, which promotes amyloid-β aggregation and accumulation in the brain, thereby exacerbating both mood and sleep disturbances as part of a deleterious feedback loop [42]. Conversely, poor sleep itself chronically activates the hypothalamic-pituitary-adrenal axis. This leads to glucocorticoid resistance, increased sympathetic nervous system activity, and higher circulating catecholamine levels, all of which exacerbate anxiety and depression [43].

The number of awakenings is a critical indicator for evaluating sleep quality [30]. In older adults, frequent awakenings not only disrupt sleep architecture but also contribute to daytime fatigue, cognitive decline, and mood fluctuations [19]. Our findings revealed that each additional awakening was associated with a 3.33-fold increase in the risk of preoperative sleep disorders among older adults, consistent with previous research [44]. Awakenings in the surgical context differ from those in chronic illness. Perioperative awakenings are often more abrupt and intense, driven by acute physiological stress and the direct effects of anesthetic and analgesic drugs [4,5]. These medications disrupt normal sleep architecture. Anesthetics, for instance, act on key neurotransmitter systems (eg, GABA, norepinephrine, and dopamine), which can dysregulate sleep-wake circuits and heighten neural sensitivity to stimuli, predisposing patients to more frequent awakenings [6,7]. This vulnerability continues after surgery, when pain, discomfort, and the hospital environment itself become primary drivers of sleep fragmentation [45,46]. Specifically, routine hospital disturbances such as nighttime noise and light can significantly impair sleep. Nocturnal light exposure is particularly disruptive, as it stimulates specialized retinal cells that signal the brain to promote arousal, thereby worsening sleep disturbances [47].

REM sleep is a critical phase, typically making up 20% to 25% of total sleep [48]. It is essential for key functions, such as memory consolidation, emotional processing, and neural recovery, helping to maintain neurotransmitter balance [49]. Our results demonstrated that each 1-unit increase in REM sleep duration corresponded to a 4% reduction in the risk of preoperative sleep disorders among older adults. Alterations in sleep architecture frequently occur with advancing age, characterized by reductions in slow-wave and REM sleep alongside increased nocturnal awakenings [3]. Older adults are predisposed to heightened anxiety and depression during hospitalization, attributable to altered sleep environments, the absence of familial support, and surgery-related psychological stress [50]. These emotional disturbances and sleep disorders interact bidirectionally, forming a vicious cycle in which anxiety and depression diminish sleep quality, which in turn exacerbates these psychological symptoms [51]. REM sleep mitigates anxiety and depression by promoting cerebral processing of emotional experiences, thereby indirectly enhancing sleep quality in older adults [52].

Light sleep typically encompasses non-REM stage 1 (N1) and non-REM stage 2 (N2) of non-REM sleep. Stage N1 represents the transitional phase from wakefulness to sleep, comprising approximately 5% to 10% of total sleep duration. In contrast, stage N2 constitutes 45% to 55% of total sleep time and represents the largest proportion of the sleep cycle [53]. Our findings indicate that each unit increase in light sleep duration corresponds to a 2% reduction in the risk of preoperative sleep disorders in older adults. During light sleep, synchronized neuronal activity facilitates the reorganization of neural networks and information processing, thereby sustaining stable brainwave patterns and enhancing cerebral sensitivity and adaptability [54]. These stages contribute distinct functions. N1 sleep, involving neurotransmitters such as dopamine, aids in processing emotional memories and can ease the transition to sleep. N2 sleep is characterized by specific brainwave patterns (sharp-wave ripples) and hippocampal reactivation, which consolidate memory by strengthening relevant neural pathways and pruning weaker ones. N2 also supports overall sleep stability, thermoregulation, and energy restoration [55]. Therefore, light sleep is not merely a structural component of sleep but a physiologically active period critical for cognitive function and emotional regulation.

### Value of a Risk Prediction Model for Preoperative Sleep Disorders in Older Adults

Clinical prediction modeling uses multifactorial models to estimate the likelihood of disease onset or the occurrence of future outcome events [56]. Wang et al [57] incorporated 9 predictors in developing a postoperative sleep disorder risk prediction model for patients undergoing arthroplasty, including preoperative sleep disorder status upon admission, wardroom type, BMI, and smoking status. Similarly, Renmei et al [58] incorporated 9 predictors, including anxiety, depression, tracheotomy, and posttraumatic stress disorder, into a risk prediction model for sleep disorders in patients discharged from the intensive care unit.

In contrast, our model is uniquely tailored to older adults in the preoperative period, a population at heightened risk due to age-related physiological changes, multimorbidity, and complex interactions between mental health and sleep [44,52]. Our model integrates 4 key predictors relevant to this group: HADS score, number of nocturnal awakenings, REM sleep duration, and light sleep duration. This selection aligns with the emphasis on psychological and sleep-architectural factors seen in previous research [24,58].

Multivariable logistic regression confirmed these as independent predictors: HADS score (OR 3.21, 95% CI 1.54-6.69), number of awakenings (OR 3.33, 95% CI 1.82-6.12), REM sleep duration (OR 0.96, 95% CI 0.93-0.99), and light sleep duration (OR 0.98, 95% CI 0.96-0.99). By incorporating objective sleep metrics, the model specifically addresses the distinct vulnerabilities of older surgical patients [59]. Calibration was strong, with close agreement between predicted probabilities and observed outcomes. DCA confirmed substantial clinical utility, showing the model provides a net benefit across a wide range of risk thresholds [32]. In summary, this model provides a novel, evidence-based tool for the early identification of older surgical patients at high risk of sleep disorders. It validates the critical role of sleep architecture and psychological state in preoperative risk. By enabling targeted interventions before surgery, it offers a proactive strategy to improve patient outcomes and potentially mitigate postoperative complications.

In clinical practice, caregivers should actively monitor the sleep health of older adults by implementing psychological interventions, such as cognitive behavioral therapy and positive thinking training; promoting family involvement; and optimizing the sleep environment and pharmacological treatments, including short-acting sedatives and anxiolytics, to enhance sleep quality and overall health [60].

For patients with severe sleep disorders, short-acting hypnotics or nonbenzodiazepine agents may be cautiously administered under clinical supervision to minimize the risk of dependence and adverse effects. On the basis of this predictive model, caregivers can devise individualized nursing interventions aimed at alleviating sleep-onset difficulties and fragmentation in older adults undergoing surgery, thereby mitigating the incidence of sleep disorders.

### Limitations

First, although the prospective cohort design of this study enhances the robustness of temporal inferences, the potential for residual or unmeasured confounding cannot be excluded. Second, the study cohort was recruited from a single tertiary hospital, which constrains the sample size and may limit the generalizability of the results to broader populations. Third, this study’s focus was confined to the immediate preoperative period, without extending follow-up into the postoperative phase. Consequently, although the model identifies presurgical risk factors, it cannot assess the temporal stability of these predictors or their longitudinal association with critical postoperative outcomes, including delirium and functional recovery. Therefore, future research should prioritize external validation across diverse clinical settings, investigation of additional potential confounders, and longitudinal assessments to elucidate how the preoperative sleep disorder risk identified by this model influences tangible postoperative outcomes.

### Conclusions

The preoperative sleep disorder risk prediction model developed for older adults in this study achieved an AUC of 0.92 (95% CI 0.88-0.96), demonstrating excellent discriminatory performance. The calibration curve indicated minimal deviation between the predicted probabilities and the observed outcomes, reflecting strong model calibration. DCA further demonstrated that the model improved net clinical benefit across risk thresholds ranging from 0.2 to 0.8, indicating high clinical utility. By integrating the quantitative relationships among HADS score, wakefulness duration, REM sleep duration, light sleep duration, and sleep disorders, the model provides a scientific foundation for the early identification and intervention of preoperative sleep disorders in older adults.

## Appendix

Multimedia Appendix 1 Clinical disease profiles of older adults.

## Abbreviations

AUC: area under the curve
DCA: decision curve analysis
GABA: γ-aminobutyric acid
HADS: Hospital Anxiety and Depression Scale
MMSE: Mini-Mental State Examination
N1: non–rapid eye movement stage 1
N2: non–rapid eye movement stage 2
NRS: Numerical Rating Scale
OR: odds ratio
PSQI: Pittsburgh Sleep Quality Index
REM: rapid eye movement
ROC: receiver operating characteristic
TRIPOD: Transparent Reporting of a Multivariable Prediction Model for Individual Prognosis or Diagnosis

## Data Availability

The datasets generated or analyzed during this study are not publicly available due to restrictions related to the source data (originating from an ongoing master’s thesis research project) but are available from the corresponding author on reasonable request.
