# Supplementary material for: Predictive Modeling of Preoperative Sleep Disorder Risk in Older Adults by Using Data From Wearable Monitoring Devices: Prospective Cohort Study
Source: JMIR Form Res. 2026 Feb 11;10:e79008. doi: 10.2196/79008 (PMC12936661; doi:10.2196/79008)
Supplement: Multimedia Appendix 1 [file formative_v10i1e79008_app1.docx]

**Table S1.** Clinical disease profiles of older adults (N=242).^a^

| Variable | | Total patients (N=242) | Patients in the non–sleep disorder group (n=202) | Patients in the sleep disorder group (n=40) | Statistics | *P* value |
| --- | --- | --- | --- | --- | --- | --- |
| **Psychometric scale scores, mean (SD)** | | | | | | <.001 |
|  | Pittsburgh Sleep Quality Index score | 7.31 (2.38) | 6.84 (2.18) | 9.65 (1.97) | –7.57^c^ |  |
|  | Hospital Anxiety and Depression Scale score | 7.21 (1.84) | 6.79 (1.64) | 9.30 (1.24) | –10.99^c^ |  |
|  | Numeric rating scale for pain score | 1.83 (1.07) | 1.62 (0.98) | 2.85 (0.92) | –7.29^c^ |  |
|  | Mini-Mental State Examination score | 20.45 (4.65) | 21.75 (3.31) | 13.93 (4.96) | 9.56^c^ |  |
| **Sleep parameters, mean (SD)** | | | | | | |
|  | Total sleep duration (min) | 473.36 (146.06) | 508.15 (130.56) | 297.62 (77.70) | 9.85^c^ | <.001 |
|  | REM^b^ sleep duration (min) | 84.25 (39.42) | 90.93 (38.74) | 50.52 (21.70) | 9.22^c^ | <.001 |
|  | Deep sleep duration (min) | 128.50 (59.70) | 138.26 (58.77) | 79.22 (35.25) | 8.51^c^ | <.001 |
|  | Light sleep duration (min) | 262.57 (92.63) | 280.75 (88.78) | 170.75 (45.12) | 11.60^c^ | <.001 |
|  | Number of awakenings | 2.68 (1.77) | 2.31 (1.50) | 4.53 (1.91) | –6.93^c^ | <.001 |
|  | Proportion of REM sleep | 0.18 (0.07) | 0.18 (0.07) | 0.18 (0.06) | 0.12^c^ | .90 |
|  | Proportion of deep sleep | 0.27 (0.09) | 0.27 (0.08) | 0.28 (0.09) | –0.29^c^ | .77 |
|  | Proportion of light sleep | 0.55 (0.10) | 0.55 (0.10) | 0.55 (0.11) | –0.00^c^ | >.99 |
| **Vital signs, mean (SD)** | | | | | | |
|  | Systolic blood pressure (mm Hg) | 130.73 (19.71) | 130.57 (19.52) | 131.53 (20.86) | –0.28^c^ | .78 |
|  | Diastolic blood pressure (mm Hg) | 78.79 (12.37) | 78.70 (12.19) | 79.25 (13.40) | –0.26^c^ | .80 |
|  | Respiratory rate (breaths per min) | 19.27 (1.29) | 19.26 (1.32 | 19.30 (1.16) | –0.17^c^ | .87 |
|  | Heart rate (beats per min) | 78.90 (12.54) | 79.07 (11.85) | 78.05 (15.76) | 0.39^c^ | .70 |
|  | Oxygen saturation (%) | 0.97 (0.02) | 0.97 (0.01) | 0.97 (0.02) | 1.01^c^ | .31 |
| **Hematologic parameters, mean (SD)** | | | | | | |
|  | White blood cell count (×10^9^/L) | 6.67 (3.27) | 6.71 (3.50) | 6.45 (1.75) | 0.46^c^ | .65 |
|  | Neutrophil percentage (%) | 0.65 (0.12) | 0.65 (0.12) | 0.66 (0.11) | –0.66^c^ | .51 |
|  | Red blood cell count (×10^12^/L) | 4.18 (0.63) | 4.17 (0.61) | 4.20 (0.70) | –0.29^c^ | .78 |
|  | Hemoglobin (g/dL) | 123.70 (19.10) | 123.82 (18.89) | 123.12 (20.37) | 0.21^c^ | .84 |
|  | Hematocrit (%) | 0.38 (0.05) | 0.38 (0.05) | 0.38 (0.06) | 0.00^c^ | >.99 |
|  | Platelet count (×10^9^/L) | 216.14 (68.70) | 217.82 (70.01) | 207.68 (61.73) | 0.85^c^ | .40 |
|  | Prothrombin time (s) | 10.65 (0.97) | 10.61 (0.94) | 10.81 (1.13) | –1.19^c^ | .24 |
|  | Activated partial thromboplastin time (s) | 27.05 (3.31) | 27.08 (3.39) | 26.91 (2.90) | 0.30^c^ | .76 |
|  | Fibrinogen (g/L) | 3.56 (1.20) | 3.59 (1.21) | 3.43 (1.19) | 0.77^c^ | .44 |
| **Biochemical parameters, mean (SD)** | | | | | | |
|  | Serum potassium (mmol/L) | 3.92 (0.39) | 3.92 (0.39) | 3.93 (0.40) | –0.13^c^ | .90 |
|  | Serum sodium (mmol/L) | 140.16 (2.32) | 140.18 (2.25) | 140.07 (2.70) | 0.26^c^ | .79 |
|  | Serum calcium (mmol/L) | 2.28 (0.12) | 2.28 (0.12) | 2.28 (0.11) | 0.23^c^ | .82 |
|  | Total protein (g/L) | 66.14 (8.58) | 66.06 (8.82) | 66.51 (7.37) | –0.30^c^ | .76 |
|  | Cholinesterase(kU/L) | 7.04 (1.84) | 7.13 (1.83) | 6.59 (1.83) | 1.71^c^ | .09 |
| **Hepatic and renal function biomarkers, median (IQR)** | | | | | | |
|  | Alanine aminotransferase(U/L) | 17.00 (12.00-25.75) | 17.00 (12.00-25.00) | 18.00 (13.75-27.25) | –0.66^d^ | .51 |
|  | Aspartate aminotransferase(U/L) | 22.00 (19.00-28.00) | 22.00 (19.00-29.00) | 22.00 (18.00-27.00) | –0.88^d^ | .38 |
|  | Prealbumin (mg/dL) | 211.00(163.75-256.75) | 211.00 (163.00-257.00) | 211.00(176.75-249.00) | –0.13^d^ | .90 |
|  | Blood urea nitrogen(mmol/L) | 5.66 (4.48-7.12) | 5.58 (4.45-7.12) | 5.79 (4.86-7.11) | –0.49^d^ | .62 |
|  | Creatinine (μmol/L) | 75.00 (62.25-93.50) | 75.00 (62.00-92.00) | 85.00 (65.75-98.25) | –1.48^d^ | .14 |
| **Type of surgical specialty, n (%)** | | | | | —^e^ | .12 |
|  | Hepatobiliary and pancreatic surgery | 13 (5.4) | 9 (4.5) | 4 (10.0) |  |  |
|  | Orthopedic surgery | 95 (39.3) | 78 (38.6) | 17 (42.5) |  |  |
|  | Urology | 56 (23.1) | 52 (25.7) | 4 (10.0) |  |  |
|  | General surgery | 36 (14.9) | 31 (15.3) | 5 (12.5) |  |  |
|  | Burn surgery | 16 (6.6) | 13 (6.4) | 3 (7.5) |  |  |

^a^Continuous variables conforming to a normal distribution are expressed as mean (SD), whereas those not conforming to a normal distribution are presented as median (IQR).

^b^REM: rapid eye movement.

^c^Independent two-sample t-test.

^d^Mann-Whitney U test (reported as Z score).

^e^Not applicable.
